# Male Red-crested Cardinal plumage coloration is associated with parental abilities and breeding performance

**DOI:** 10.1038/s41598-019-47498-6

**Published:** 2019-07-29

**Authors:** Luciano N. Segura, Bettina Mahler

**Affiliations:** 1 Sección Ornitología, División Zoología Vertebrados, Museo de La Plata, Universidad Nacional de La Plata, Paseo del Bosque s/n (B1900FWA), La Plata, Argentina; 2 IEGEBA, CONICET-UBA, Departamento de Ecología, Genética y Evolución, Facultad de Ciencias Exactas y Naturales, Universidad de Buenos Aires, Pabellón II Ciudad Universitaria (C1428EGA), Buenos Aires, Argentina

**Keywords:** Behavioural ecology, Animal behaviour

## Abstract

Avian plumage coloration deriving from carotenoid-based pigments is among the most honest signals of individual quality. It has been argued that females may differentially allocate resources based on mate attractiveness or quality, paying the costs of investing more in a current breeding attempt. We tested predictions of the differential allocation hypothesis on the natural variation of carotenoid-based plumage using the brightly red-colored head plumage of the Red-crested Cardinal (*Paroaria coronata*). It is to our knowledge the first time this hypothesis is tested on the natural variation of this pigment on a wild bird. We found that the brightness of the males’ red plumage patch is positively associated with their reproductive success and the nest defence they provide. We also found that brighter males invest less in their offspring (by delivering less food to their nestlings and poorly cleaning the nest) than duller males and, by contrast, females mated with brighter males invest more in parental care. Our results are consistent with the differential allocation hypothesis: differential allocation allowed breeding pairs with brighter males to produce more offspring, suggesting that it can be considered adaptive and should be included in studies of eco-evolutionary dynamics.

## Introduction

The expression of morphological characters that function as indicators of individual quality is well documented among animals^1^. For example, the intensity of avian plumage colouration may inform about the state of the bird’s body condition^2–4^, health and parasite resistance^2,5^, and also about its characteristics as a breeder^1^. Plumage coloration deriving from carotenoid-based pigments that are obtained from the diet^6^ has been frequently mentioned as an honest indicator of individual quality^7–9^. Weaver *et al*.^4^ recently performed a meta-analysis of published studies on the relationship between carotenoid-based feather coloration and measures of individual quality, and confirmed that, in general, feather coloration is an honest signal of quality. Moreover, since birds with red carotenoid-based coloration only ingest yellow carotenoid pigments from the diet (e.g., lutein and zeaxanthin) and they have to bio-convert yellow pigments to red in an even more costly process^10^, these authors also showed that converted, but not dietary, carotenoids drive the relationship between feather coloration and individual quality. Individual quality in birds can be translated as the ability to obtain food, but also as the metabolic capacities of absorption, transformation and fixation of these pigments in the feathers^4,11^. Since carotenoid-based pigments are essential in cellular processes such as immuno-stimulation^6^, antioxidants^12,13^, deactivation of free radicals^14^ or metabolism of vitamin A^15^, only those individuals in better body condition manage to accumulate a greater proportion of pigments in the feathers^16–18^.

Whenever offspring reproductive value is directly related to the attractiveness of the mate, the ‘differential allocation hypothesis’ receives special interest^19–21^. This hypothesis especially applies to socially monogamous species with biparental care and carotenoid-based plumage^21^, and predicts that females should be willing to pay the costs of investing more in the current breeding attempt when mated to attractive males^19^. As a consequence, males may reduce their effort in nest attention. Several experimental studies in birds have found support for the differential allocation hypothesis^20–24^. Although with robust and widely accepted results, all these studies have either removed or covered the trait analysed rather than showing an effect of natural signal variation^25,26^. To our knowledge, there are no studies that focus on the natural variation of carotenoid-based plumages as traits that can function as indicators of quality to shed light on this mechanism in the context of sexual selection.

The Red-crested Cardinal (*Paroaria coronata*) is, from an avian visual perspective, a sexually dichromatic neotropical bird with a conspicuous red crest on its head (Machado and Segura, *unpubl. data*). This species shows a high level of natural variation in the intensity of the color of its crest (Machado and Segura, *unpubl. data*, also see ref.^27^). Thus, Red-crested Cardinals provide a good model to test the differential allocation hypothesis in a natural context instead of artificially manipulating the variation of the red colour intensity. If high-quality Red-crested Cardinal males can afford to extract, metabolize and deposit more carotenoids in their plumage, then these individuals will also be the most intensely coloured ones^7^. We studied Red-crested Cardinal breeding pairs in a natural nesting habitat and obtained data of the main reproductive parameters and of the males’ plumage coloration. We tested the following predictions derived from the differential allocation hypothesis: 1) breeding pairs with more attractive males (i.e., with a more intense red plumage) will have higher breeding success (i.e., higher proportion of successful nests and higher egg and nestling survival) than breeding pairs with less attractive males; 2) more attractive males will invest less in parental care (i.e., lower intensity of nest defence and lower feeding and faecal extraction rates) than less attractive males; and 3) females mated with more attractive males will invest more in parental care (i.e., higher intensity of nest defence and higher feeding and faecal extraction rates) than females mated with less attractive males.

## Results

### General breeding parameters

Before the start of the breeding season we captured 50 (22 males), 42 (23 males) and 51 (27 males) individuals in 2011, 2012 and 2013, respectively. We marked captured individuals with colour bands (see Methods) to allow future identification of the adults. We found a total of 9, 10 and 11 previously captured males nesting during the breeding seasons in each of the years of this study. We were able to follow 26 of these males throughout the entire length of the breeding season; 11 pairs where both male and female were banded and 15 pairs where only the male was banded. Each breeding pair had 4.63 ± 0.26 breeding attempts per season (range = 2–7). Of all nests monitored (n = 117, this included nests with marked and unmarked individuals), 23% were successful, 69% were depredated, 5% were abandoned as a result of botfly parasitism and 3% failed due to unknown causes.

Plumage brightness and hue were correlated; thus, the latter parameter was not used for analyses. Colouration was independent of males’ body size (mean tarsus length 27.85 ± 0.17; range = 26.4–29.9) and condition (mean weight 51.79 ± 0.81 g; range = 44.2–59.5), showing no significant correlation for neither brightness (body mass: r = 0.02, P = 0.93; tarsus: r = 0.21, P = 0.34) or red chroma (body mass: r = −0.12, P = 0.57; tarsus: r = −0.15, P = 0.48; n = 26).

**Figure 1:**
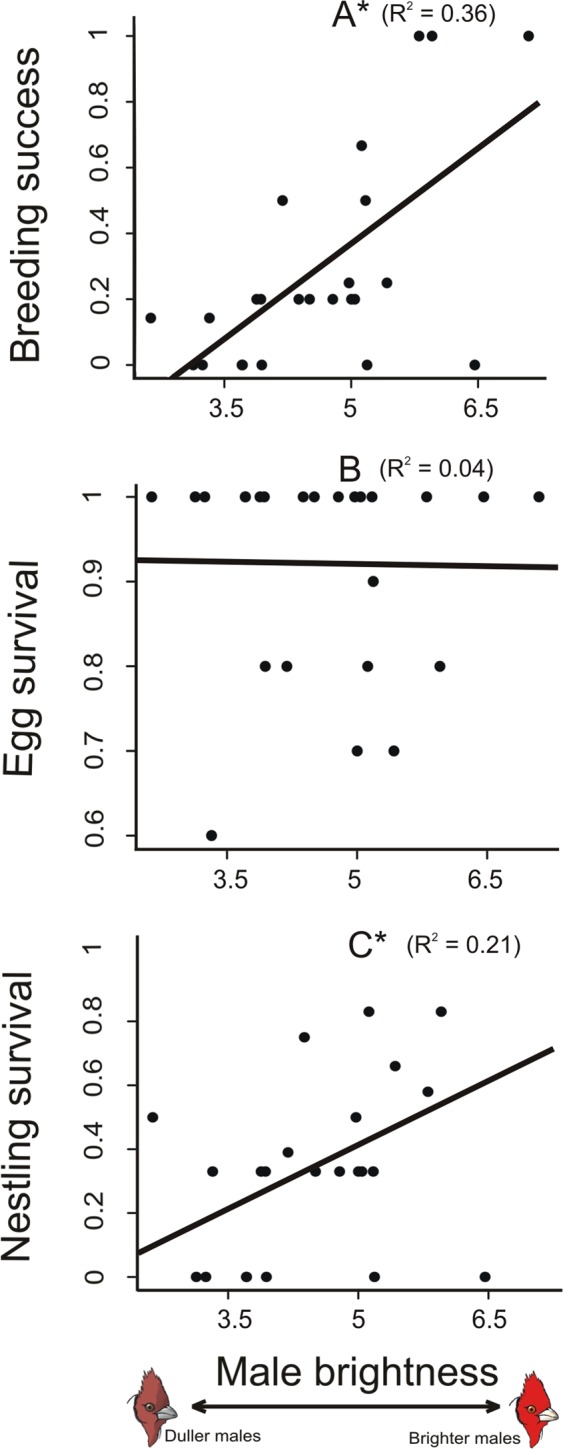
Brightness of males and general reproductive success. Relationship between the brightness of the males’ red plumage patch and the proportion of successful nests throughout the breeding season (**A**), egg survival rate (**B**) and nestling survival rate (**C**). Asterisks indicate significant relationships. Figures show raw data and lines represent simple regressions between pairs of variables.

For each pair, reproductive success was calculated by estimating the overall breeding success throughout the breeding season (i.e., the proportion of successful nests in a season), egg survival and nestling survival. In general, the brightness of the male’s red plumage patch, but not its chroma, was correlated with their reproductive success (Table 1). We found that brighter males had a higher proportion of successful nests throughout the breeding season and higher nestling survival rates (Table 1, Fig. 1). The positive linear relationships (Table 1) showed that an increase of only 1% in male average reflectance increased 31% the breeding success and 17% the nestling survival of the breeding pair.

**Table 1:** Effect of male coloration on the breeding success of Red-crested Cardinals breeding pairs.

	*df*	Male brightness			Male red chroma		
		*Estimate*	*t*	P	*Estimate*	*t*	P
Breeding success	23	**0.31**	**2.13**	**0.03**	2.59	0.45	0.68
Egg survival	39	0.02	1.06	0.32	0.16	0.37	0.21
Nestling survival	29	**0.17**	**3.21**	**<0.01**	2.98	1.66	0.09

Effect of male brightness and male red chroma on the breeding success (proportion of successful nests throughout the breeding season), egg and nestling survival of breeding pairs of Red-crested Cardinals. Breeding success was estimated from 26 banded pairs, egg survival from 42 nests and nestling survival from 32 nests.

We also analysed nestling growth (body mass and tarsus length) using nonlinear mixed models (see Methods). Male brightness was associated with nestling growth, both for nestling size and mass (Table 2). The maximum growth rate of nestlings of brighter males was lower and occurred later during the growth period than that of nestlings of duller males; however, final nestling size (asymptote) was not correlated with male plumage coloration (Table 2). Nestlings’ growth parameters were independent of the number of nestlings at the nest (Table 2).

**Table 2:** Effect of male coloration and clutch size on the nestling Red-crested Cardinal growth parameters.

	*df*	Male brightness			Male red chroma			# Nestlings		
		*Estimate*	*t*	*P*	*Estimate*	*t*	*P*	*Estimate*	*t*	*P*
Body mass (g)										
Asymptote	139	0.86	0.82	0.41	0.18	0.89	0.39	0.07	1.25	0.21
Max growth	139	**−0.12**	**−2.27**	**0.02**	**−**0.04	1.24	0.21	0.001	**−**0.63	0.52
Day max growth	139	**0.42**	**1.89**	**0.05**	2.16	**−**1.69	0.09	0.05	1.56	0.12
Tarsus (mm)										
Asymptote	139	0.85	1.09	0.27	1.23	0.17	0.87	2.45	0.31	0.75
Max growth	139	**−0.33**	**−2.31**	**0.02**	2.46	**−**0.52	0.6	3.34	0.01	0.98
Day max growth	139	**0.4**	**3.67**	**<0.01**	0.99	0.02	0.98	1.29	1.01	0.31

Growth parameters (body mass and tarsus length) were characterized as: upper asymptote (fledging size), maximum relative growth rate and age of maximum relative growth rate. Growth parameters were estimated from 40 nestlings from 19 banded males.

### Nest defence behaviour

We tested agonistic behaviours by presenting nest predator dummy models to breeding pairs of Red-crested Cardinals that we previously banded. We conducted the experiments in 11 nests halfway through the incubation stage and we measured the following behaviours: 1) frequency of attacks to the model, 2) frequency of close passes to the model, 3) frequency of aggressive calls, and 4) proportion of time they were perched at less than 2 m away from the model. We found that brighter males were more aggressive towards an avian predator (Table 3), as they emitted calls more frequently (almost five new aggressive calls) and attacked the dummy more often (almost three new attacks) with each increase of 1% average brightness. Females of brighter males showed a slight level of aggressiveness towards the predator (Table 3), only emitting calls more frequently in the presence of the dummy model. Red chroma was not correlated with male or female nest defence behaviours (Table 3).

**Table 3:** Effect of male coloration on parental nest defence behaviour of Red-crested Cardinals.

	*df*	Male brightness			Male red chroma		
		*Estimate*	*F*	P	*Estimate*	*F*	P
Male defence							
Aproach (%)	9	34.98	3.06	0.12	40.5	0.21	0.66
Calls (#)	9	**4.64**	**6.98**	**0.03**	12.91	0.33	0.58
Passes (#)	9	0.52	0.83	0.39	18.98	0.99	0.35
Attacks (#)	9	**2.59**	**9.49**	**0.01**	**−**5.24	0.11	0.74
Female defence							
Aproach (%)	9	16.83	0.42	0.53	53.12	0.59	0.46
Calls (#)	9	**4.18**	**6.36**	**0.04**	**−**40.4	0.22	0.65
Passes (#)	9	**−**0.12	0.98	0.35	10.01	0.99	0.35
Attacks (#)	9	0.24	0.81	0.39	3.91	0.13	0.72

Effect of male brightness and male red chroma on the agonistic responses of breeding Red-crested Cardinals to the presence of a dummy predator model close to the nest. Overall models did not show significant relationships between male coloration and defence response, neither for males (male brightness: Wilks’ lambda = 0.35, P = 0.28; male red chroma: Wilks’ lambda = 0.36, P = 0.3), nor females (male brightness: Wilks’ lambda = 0.31, P = 0.23; male red chroma: Wilks’ lambda = 0.55, P = 0.22).

### Nestling attendance

**Figure 2:**
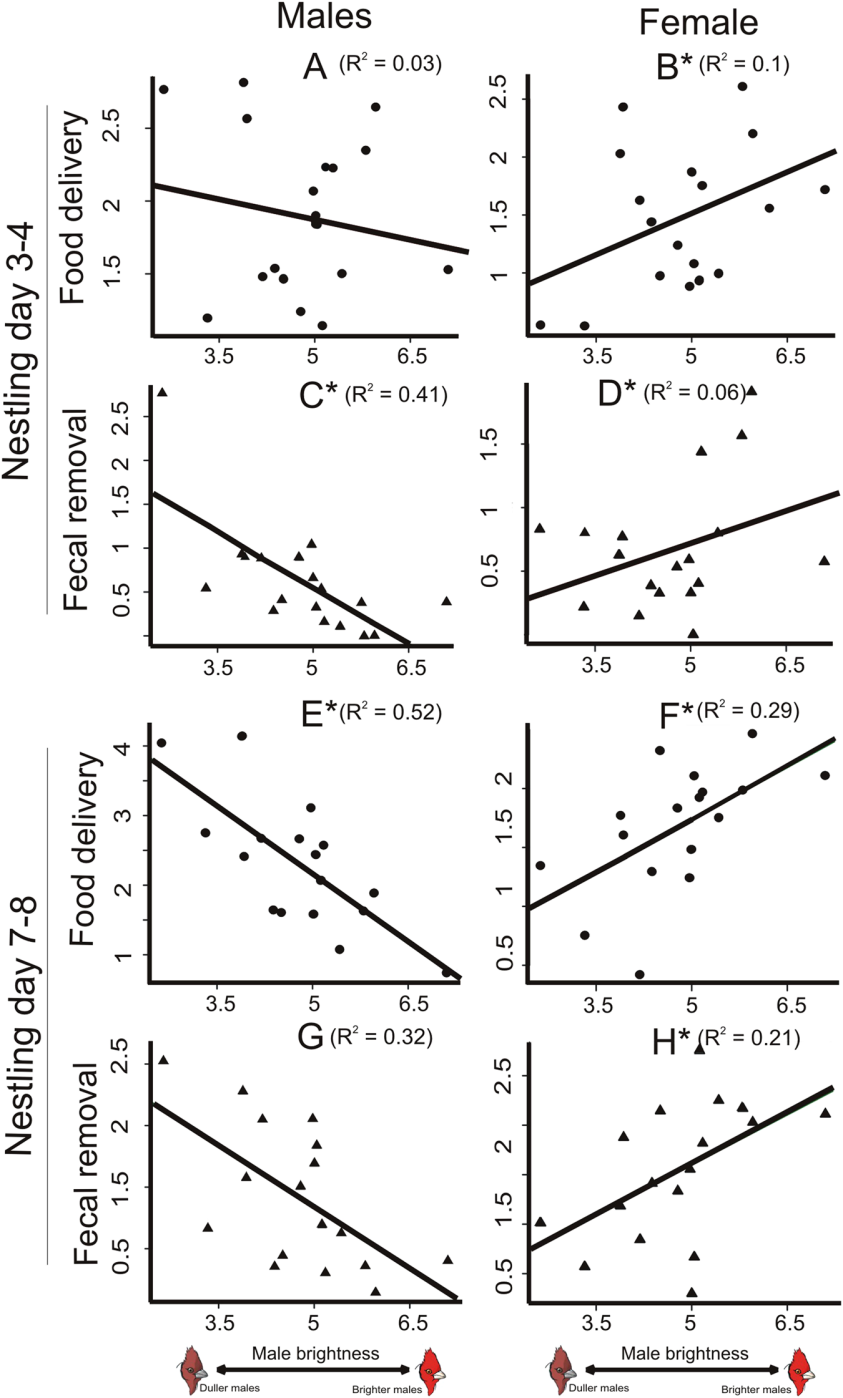
Brightness of males and parental nestling attendance. Male and female feeding delivery (circles) and faecal sacs removal (triangles) in relation to the brightness of the males’ red plumage patch. (**A**–**D**) show parental effort on nestling days 3–4 and (**E**–**H**) on nestling days 7–8. Asterisks indicate significant relationships. Figures show raw data and lines represent simple regressions between pairs of variables.

We documented rates at which Red-crested Cardinal breeding pairs provisioned nestlings and eliminated faecal sacs at two points during the nestling stage: first on nestling day 3–4 and again on nestling day 7–8. We found that brighter males invested less in their offspring than duller males. Male deliveries to the nestlings (standardized per hour and nestling) on nestling day 3–4 were not associated with male brightness or red chroma, whereas on nestling day 7–8 they were negatively associated with male brightness (Table 4, Fig. 2). The negative linear relationships (Table 4) showed that an increase of 1% in male average brightness impacted in one less male food delivery per hour to each nestling. Male removal of the nestlings’ faecal sacs was also negatively associated with male brightness on nestling day 3–4 (Table 4, Fig. 2). We also recorded the volume of food provisioned to the nest for males and females, separately. Male plumage coloration was not associated with the volume of food they delivered (Table 4).

**Table 4:** Effect of male coloration on parental effort of breeding Red-crested Cardinals.

	*df*	Male brightness			Male red chroma		
		*Estimate*	*F*	P	*Estimate*	*F*	P
Nestling day 3–4							
Male food delivery	16	**−**0.43	2.09	0.17	4.91	0.27	0.61
Female food delivery	16	**0.89**	**5.83**	**0.03**	0.09	0.00	0.99
Male faecal removal	16	**−0.59**	**8.23**	**0.02**	1.14	0.02	0.88
Female faecal removal	16	**0.66**	**6.04**	**0.03**	1.95	0.04	0.84
Male food volume (%)	16	0.02	0.69	0.42	**−**0.05	0.31	0.58
Female food volume (%)	16	0.04	1.87	0.19	0.29	0.1	0.75
Nestling day 7–8							
Male food delivery	15	**−1.1**	**4.94**	**0.04**	**−**0.21	0.001	0.97
Female food delivery	15	**1.33**	**8.29**	**0.01**	1.78	0.011	0.91
Male faecal removal	15	**−**0.39	1.88	0.19	10.63	0.63	0.44
Female faecal removal	15	**0.41**	**4.89**	**0.04**	0.05	0.001	0.98
Male food volume (%)	15	0.01	0.03	0.85	0.54	0.31	0.59
Female food volume (%)	15	**0.06**	**10.03**	**0.006**	0.11	0.02	0.88

Effect of male brightness and male red chroma on feeding rates, faecal sacs removal rates and volume of food provisioned to the nest at two points during the nestling stage: nestling day 3–4 and nestling day 7–8. Overall models did not show significant relationship between male coloration and parental effort on nestling day 3–4 (male brightness: Wilks’ lambda = 0.37, P = 0.1; male red chroma: Wilks’ lambda = 0.85, P = 0.94) nor on nestling day 7–8 (male brightness: Wilks’ lambda = 0.31, P = 0.06; male red chroma: Wilks’ lambda = 0.85, P = 0.93).

On the other hand, females of brighter males invested more in their offspring than females of duller males. Female deliveries to the nestlings and removal of the faecal sacs were positively associated with male brightness both for nestling day 3–4 and 7–8 (Table 4, Fig. 2). Females of brighter males also delivered larger food items on nestling day 7–8 (Table 4). For these females, the positive linear relationships (Table 4) showed that an increase of 1% in male average brightness was related to an increase in prey-items delivered per hour to each nestling, in addition to a 6% increase in the size of food they delivered.

## Discussion

Our results show that breeding pairs with brighter males had higher nesting success throughout the breeding season and higher nestling survival rates in relation to breeding pairs with duller males. Our models showed that increased male average brightness significantly impacted on the reproductive fitness of the breeding pairs. The combination of higher breeding success and higher nestling survival directly results in a higher fledgling recruitment in the population by the brightest males. Similarly, other studies have found that more attractive males produced more fledglings during a breeding season [i.e., ref.^28^ for Yellowhammers (*Emberiza citrinella*), ref.^29^ for House Finches (*Carpodacus mexicanus*), or ref.^30^ for Middle Spotted Woodpeckers (*Dendrocopos medius*), but see ref.^31^ for American Redstarts (*Setophaga ruticilla*)]. Recently, Laczi *et al*.^32^ found that a reliable male quality indicator (i.e., the white forehead patch size of the Collared Flycatchers, *Ficedula albicollis*) was also associated with a higher number of fledglings. Our results indicate that, in accordance with our first prediction, the red crest coloration of our study species is a reliable indicator of quality, which reinforces the idea that carotenoid-based plumage is an honest indicator of quality among birds. In this context, the brightness of the males’ red head may have an important role in intersexual signalling, although further research is needed to understand female mate choice in this system.

Nestling growth was also associated with male brightness, but strikingly (in light of the higher nestling survival of brighter males), we found an inverse relationship. The maximum growth rate of nestlings of brighter males was not only smaller, but also delayed in time. We found that female feeding rates increase at days 7–8, but male delivery rate might decrease before females start compensating (between days 3–4 and 7–8) thus occasioning lower growth rates. Both parameters (maximum growth rate and age of maximum growth) would suggest that females are not fully able to compensate for the male’s reduced feeding rate. However, these remissions in terms of growth were not reflected in the asymptotic values of body mass and size at the time of fledging, which are good estimators of nestlings’ survival^32^. It has been previously argued^33,34^ that more intensely coloured males (i.e., carriers of good genes) may confer a metabolic growing advantage to their offspring. Although our results cannot confirm this idea, offspring of high quality males (i.e. brighter males) might have some intrinsic characteristics to better metabolize food and be able reach the same body mass and size at the time of fledging than offspring of duller males. In this sense, Laczi *et al*.^33^ recently found a significant genetic relationship between male ornamentation and the resource assimilation capacity of the nestlings (also see refs.^35,36^).

Our results partially supported the second prediction, i.e. that brighter males would invest less in parental care. On one hand, contrary to our prediction, we found that brighter males made a higher number of direct attacks to the predator model and also emitted calls more frequently when they were exposed to the dummy. Similarly, Pryke *et al*.^25^ found that males of the Red-collared Widowbirds (*Euplectes ardens*) with stronger (larger and redder) carotenoid signals defended their nesting territories more effectively, suggesting that carotenoid-based colorations are good indicators of dominance and fighting ability^37^ (but see ref.^38^). As nest predation is a key determinant of fitness for the Red-crested Cardinal during the breeding season^39–41^, effective brood defence will significantly decrease nest failure^38,42^. Our data suggest that in the Red-crested Cardinal, red carotenoid-based coloration is an honest signal of male fighting ability. As such, the nest defence provided by a high quality male may be a reliable paternal ability, which a female could focus on during mate selection. High levels of testosterone were shown to be related to male ornamentation and aggressive behaviours^43^. Although we did not measure testosterone levels, we cannot rule out the possibility that the positive relationship between nest defense and brightness is intimately linked to this hormone^43,44^.

On the other hand, brighter males cleaned the nest less often and delivered less food to the nestlings in relation to duller males, providing support to our second prediction. Although some studies have failed to find consistent relationships between male ornamentation and their own nestlings attendance^32,45^, we corroborated that brighter males provisioned their own nestlings at lower rates. Previous studies suggest that for high-quality males, displaying a more conspicuous coloration could be associated with increased costs of feather-wear^46,47^, so more attractive males would provide less food to their nestlings^48,49^. But despite this pattern, brighter males showed higher nestling survival rates and a larger proportion of successful nests throughout the breeding season (also see ref.^28^), and nestlings also reached an acceptable asymptotic value of body mass and size at the time of fledging. While, as discussed above, this could be related to a heritable advantage to better metabolize the resources under food stress, it might also be related to the quality of food items provided to the chicks. However, we did not find differences between males in the volume of prey delivered.

Finally, chick growth could be explained by female behaviour, as suggested by our third prediction. Female feeding rate and nest faecal sacs removal rate were positively related to male brightness. However, similar to the males’ behaviour discussed above, females mated with brighter males did not show higher nest defence, suggesting that this trait could be a fixed male quality attribute instead of a direct effort in relation to the current breeding attempt. According to Burley’s^19^ original presentation of the differential allocation hypothesis, we found an increase in offspring attendance of females mated to attractive males. Our results support the idea that females are increasing their effort in offspring attendance (both higher food delivery rates and prey volume) at the expense of maintaining a high quality mate. Similarly, Dakin *et al*.^50^ reported that female Tree Swallows (*Tachycineta bicolor*) that were paired to more attractive males (in this case, a blue-green structurally coloured plumage) fed offspring at higher rates. In the context of the differential allocation hypothesis, Sheldon^21^ proposed that attractive mates must provide environments that are particularly suitable for the development of offspring. In our study, this advantageous environment was represented by the more intense nest defence provided by brighter males. Thus, if a nest is safe due to an effective male defence, the female can increase her effort delivering more food to the nestlings^51^.

Within the framework of the differential allocation hypothesis, there are at least two factors that could be limiting our results. On one hand, brighter males could have such food-rich territories that it comes at no extra cost to the female to increase her share of care relative to females on poorer territories^21^. In this sense, more connected forest patches with tall trees provide more refuge to nests and higher food supply for Red-crested Cardinal breeding pairs (LNS, *upubl. data*). However, the spatial distribution of the males in our study site did not show a pattern associated to territory quality, with brighter males being randomly distributed across the study site (LNS, *pers. obs*.). On the other hand, we cannot completely rule out the possibility that our results were confounded by the age of individuals. The age of the individuals could be linked to the intensity of plumage colouration^17^, and this in turn be linked to the experience as a breeder. Nonetheless, most of the monitored males in our study (~90%) were not first-year breeding males, which are easily recognizable by the small remnants of brown feathers on their crest. Thus, the likelihood of this effect altering our results is neglectable.

For the first time, we showed partial support for the differential allocation hypothesis in a study system with natural variation of an individual quality trait based on carotenoid pigments. While, as predicted, females invested more when paired to brighter males, these did not decrease their defence behaviour, although they invested less in their offspring. Our results provide evidence in favour of the theoretical prediction that females should invest more in reproduction when mated with high-quality males. Greater female investment in terms of feeding rate and nest sanitation when paired with attractive males implies that studies that do not estimate or control for maternal investment will tend to overestimate the direct and indirect benefits of male attractiveness on offspring viability^26,52^. In this way, some breeding advantages only attributed to male contribution (i.e., ‘good genes’) could actually be driven by differential female allocation^53^. In light of our results, females are gaining direct (higher nest defence, overall nesting success or nestling survival) and indirect (potential ‘good genes’ for their offspring) benefits when mated to attractive males, and breeding pairs with attractive males are producing more offspring. Then, differential allocation should effectively be considered adaptive^54^ and included in studies of eco-evolutionary dynamics.

## Methods

### Species and study site

The Red-crested Cardinal is, from a human perspective, a sexually monochromatic species that inhabits semi-open areas with scattered trees and shrubs^55,56^ from east central Argentina to southern Brazil, Paraguay, eastern Bolivia, and Uruguay. Recently, Machado and Segura (*unpubl. data*) found that this species is sexually dichromatic from a bird perspective, mainly in plumage brightness. Our study site (35°20′S, 57°11′W) is a flat area of semi-open grasslands with several low chains of woodlands dominated by native tree species. In this site, Red-crested Cardinals breed from early October to late February. They build open-cup nests and each breeding pair has 4–5 nesting attempts over the breeding season, modal clutch size is three eggs, and each nest has an estimated chance of survival of 14%^40,57^.

### Capture of birds, sex determination and reflectance spectrometry

During three consecutive years (2011–2013), from August to September (before the start of the breeding season), we captured adult individuals with mist-nets in wintering communal feeding sites. Individuals were colour-banded with a unique combination to establish the identity of breeders during reproductive attempts and following variables were measured: 1) mass (g), 2) tarsus length (mm), and 3) reflectance of the red plumage. Additionally, to estimate bill volume (see below), we measured: 1) distance from the most proximal edge of the commissure to the bill tip measured on the right side of the bill, 2) distance between most proximal points of the commissure, and 3) vertical distance from the top of the maxilla to the bottom of the lower mandible measured at the base of the bill.

We also took a small amount (15–30 mL) of blood for sex determination through brachial vein puncture with a 31G needle. Blood collection, sample conservation, and DNA extraction procedures are detailed in De Mársico *et al*.^58^. Individuals were genetically identified as male or female following Griffiths *et al*.^59^.

We quantified plumage reflectance with an Ocean Optics S2000 spectrometer with a PX-2 pulsed xenon light source and a bifurcated fiberoptic probe (Ocean Optics, Inc.). We calibrated measures of reflectance against a white standard of barium sulfate and against a black standard^60^. The probe was housed in a black plastic tube to minimize incident ambient light. We kept the distance between the probe and the body surface constant (17.05 ± 0.1 mm). The angle of incidence of illumination and reflected light measured was 90° to the feather surface. During the first captures (2011–2012), we measured plumage coloration of 15 individuals both directly on the bird in the field and from collected feathers in the laboratory. In the field, we measured a homogeneously coloured red region located immediately behind the erected crest. The red patch size allowed us to take four different reflectance measures. We then collected about 12–15 feathers from each bird from the same area. Once in the laboratory, we superimposed the collected feathers following Quesada and Senar^61^ on a dark velvet surface, trying to imitate the plumage surface of the bird. Over the superimposed feathers, we took four different reflectance measures. Both reflectance measures were highly correlated (Spearman’s rank correlation: r = 0.89, P < 0.001), so we collected the feathers for the rest of the captures and took the reflectance measures in the laboratory following the above-mentioned procedure. In all cases, we took each measurement within a diameter of 6 mm and recorded reflectance from 300 to 700 nm in 0.35-nm steps. We recorded data via the spectral-acquisition software package OOIBASE32 (Ocean Optics, Inc.). The procedure for reflectance data collection (including the elimination of artifactual “spikes”) is described in Facchinetti *et al*.^62^. We analysed reflectance spectra by calculating brightness (the average reflectance between 300 and 700 nm), hue (the wavelength at which reflectance is halfway between its minimum and its maximum) and red chroma (calculated as the ratio between the red region’s reflectance, 580 nm–700 nm, and total reflectance)^63^. Brightness was significantly correlated with hue (r = −0.7, P = 0.02) but not with red chroma (r = −0.22, P = 0.29). We therefore only used brightness and red chroma in the analyses.

### Nest monitoring

We collected data during three consecutive breeding seasons, from October to February 2011–2014. During each breeding season, immediately after capture and banding, we monitored the nests of pairs where at least the male was banded. Each breeding pair was monitored in only one breeding season. We found nests by systematically searching potential nest sites and observing the behaviour of territorial pairs. As at least one member of the pair was banded, we are confident that we assigned all nests to each pair throughout the breeding season. We visited nests daily during egg-laying and at the time of hatching, and every two days during the incubation and nestling stages. Eggs and nestlings within the nest were marked with waterproof ink for individual identification. On each visit, we weighed nestlings to the nearest 0.2 or 0.6 g using Pesola spring balances of 20 and 60-g capacity, respectively, and measured tarsus length to the nearest 0.05 mm with a Vernier calliper. After nestlings were 12 days old, we inspected nests from a distance of 1–3 m to minimize the risk of premature fledging. We checked nests until they failed or young fledged. We considered a nest deserted if eggs were cold and no parental activity was observed near the nest during the visit (i.e., 15–20 min), or when all chicks died as a result of botfly (*Philornis* spp.) parasitism^64^. We considered a nest predated if nest contents disappeared between consecutive visits and there was no parental activity near the nest.

### Breeding parameters and nestling growth rate

For each pair, we estimated overall breeding success throughout the breeding season as the proportion of nests that produced at least one fledgling. Reproductive success was also estimated using two standardized parameters: (1) egg survival, calculated as the proportion of eggs that survived until the end of the incubation in nests that survived until the nestling stage and (2) nestling survival, calculated as the proportion of nestlings that fledged from those that hatched in nests that survived the entire nesting cycle. We analysed the data using generalized linear mixed models (glmm)^65^, where breeding success, egg and nestling survival were included as response variables, male brightness and red chroma as predictor variables, and breeding pair as a random variable.

We analysed chick growth using nonlinear mixed models^66^. Nestling growth data (body mass and tarsus length) were fitted to Richards equation^67^ using the parameterization proposed by Tjørve and Tjørve^68^ (also see ref.^69^ for details). Brightness and red chroma were included as predictor variables for each growth parameter: upper asymptote (fledging size), maximum relative growth rate and age of maximum relative growth rate. Positive estimates were interpreted as positive relationships between explanatory and response variables, or vice versa^67^. We included nestling identity and nest as random effects. Since two breeding pairs managed to reproduce successfully twice in a single season, we also included breeding pair as a random effect. Statistical analyses were carried out using packages from statistical software R^65^, including nlme^70^.

### Nest defence behaviour

We tested agonistic behaviours by presenting dummy models (taxidermic mounts in a life-like position) to banded breeding pairs. Nests were tested with three different models (used alternately) of male Guira Cuckoos (*Guira guira*). Guira Cuckoos are sympatric with Red-crested Cardinals, are considerably larger (Red-crested Cardinal body mass: 45 g; Guira Cuckoo body mass: 150 g) and are nest predators during the egg and early nestling stages. Experiments were restricted to the predators, since Segura and Reboreda^71^ showed that Red-crested Cardinals responded aggressively towards the predator control dummy model of the Guira Cuckoo but did not have aggressive behaviours towards a control dummy model (Chalk-browed Mockingbirds, *Mimus saturninus*). We conducted the experiments in 11 nests during middle incubation stage, early in the morning (07:00–10:00). We attached the model to a branch ~0.8 m far from the nest at the same height and pointing to it. Each trial began when the pair returned to the nest (in all cases they returned together) and lasted for 5 min. We video recorded (Handycam Sony HDR-CX330) agonistic behaviours with a video camera placed 10–15 m far from the nest.

We analysed the videotapes in the laboratory and determined the following behaviours: 1) frequency of attacks to the model (attacks), 2) frequency of close passes to the model (passes), 3) frequency of aggressive calls (calls), and 4) proportion of time they were perched less than 2 m far from the model (approach). We chose these variables to characterize host responses according to the level of aggressiveness or degree of risk taken (attacks > passes > calls > approach)^71^. To control for multiple comparisons and given the lack of independence between these four variables, significance of predictor variables (brightness and red chroma) was evaluated using MANOVA (Wilks’ Lambda).

### Nestling attendance

We documented rates at which males and females provisioned nestlings and eliminated faecal sacs by videotaping nests at two points during the nestling stage: first on nestling day 3–4 and again on nestling day 7–8 (n = 18 and 17 nests with captured males, respectively). We standardized the total deliveries per hour relative to the number of nestling separately for males and females. Videocameras (Handycam Sony HDR-CX330) were located 3–4 m from nests, mostly by tying it to a branch inside the tree canopy (only in 4 nests we were able to use a tripod to support the videocamara). We taped activity at nests continuously for 4–5 h, starting 70–100 min after sunrise. Before positioning the videocamaera, we first habituated the pair from the previous day with a cardboard model that simulated the videocamera. To control for hesitancy to return to nests after our presence, we began extracting data from each video recording with the first feeding visit to the nest. Volume of food provisioned was calculated for each visit by visually estimating the volume of food items relative to adult bill volume, in 10% increments (see details in Rivers^72^). Bill volume was calculated using the formula for bill volume proposed by Greenberg and Droege^73^. For males (n = 18) and banded females (n = 9), bill measurements were taken directly during captures. For non-banded females (n = 9), we used the average bill measurements of banded females. To control for multiple comparisons and given the lack of independence between these variables (food delivery rate, faecal removal rate and food volume), significance of predictor variables (brightness and red chroma) was evaluated using MANOVA (Wilks’ Lambda).

### Ethical note

This study was conducted with research permits from the regional nature conservation authority (Organismo Provincial para el Desarrollo Sostenible, *OPDS* #17717, Dirección de Áreas Naturales Protegidas, Buenos Aires province, Argentina). The experimental protocols were approved by the National University of La Plata and the Consejo Nacional de Investigaciones Científicas y Técnicas (Res. # 315114). As the experimental protocols involving the capturing and handling of birds were of minimal impact for birds, the National University of La Plata committee for animal care and use did not intervene.

## Data Availability

The datasets generated and analysed during the current study are available from the corresponding author on reasonable request.
